# Nursing during Times of Pandemic: from Courage on the Front Line to Heroic Courage in Banksy's Mural

**DOI:** 10.17533/udea.iee.v38n2e02

**Published:** 2020-07-10

**Authors:** R. Mauricio Barría P.

**Affiliations:** 1 RN, M.Sc, DrPH. Director of the Institute of Nursing, Faculty of Medicine, Universidad Austral de Chile. Chile. Email: rbarria@uach.cl Universidad Austral de Chile Universidad Austral de Chile Chile rbarria@uach.cl

On 12 May 2020, the world commemorated the bicentennial of the birth of Florence Nightingale, and to date, multiple publications in press media, social networks, and journal articles have related her biography and accounted for her contribution and legacy, not only to nursing, but to public health. It is precisely within this context of health contingency due to the COVID-19 pandemic that the relevance of Nightingale gains greater sense on aspects as simple, but as necessary, like handwashing and measures of basic health.

This year, additionally, the World Health Organization designated it as the *Year of Nursing and Midwifery*(1) and in parallel published in April the report on the State of Nursing in the World, 2020,(2) highlighting its fundamental role as part of the teams integrated to reach universal health coverage and other national and global health objectives. The report highlights that nurse’s number approximately 28 million, which represents nearly 59% of the labor force of health professionals, with such being the most-numerous groups. However, there are still sectors and settings in which an important gap of these professionals still persists to cover efficiently the demands of the population. 

Although all the aforementioned already highlights the work and role of Nursing throughout the world, it is undoubtedly this unique moment in which the international community transversally has had the opportunity to understand more broadly what health professionals mean, and specially, nurses. But nursing during pandemics already has registries, which, although at the time did not show the actions and importance of nursing, retrospectively, its value has been verified. Thus, for example, during the 1918 influenza pandemic, nurses in New York provided care to thousands of patients with minimum federal support, but working in coordination with local community agencies to establish improvised hospitals and respond to the high demand for care.(3) Most recently, during the 2009 influenza A (H1N1) pandemic, the high compromise was again found to comply with the task of care, estimating that over 90% of nurses manifested the intention of working during the pandemic and that, as it is logical to think, there was a significantly higher probability of working if they were provided with adequate personal protection equipment (PPE). In contrast, nearly 7% reported that they would not be willing to work during a flu pandemic, independent of incentives or other factors. This demonstrated that to maintain an adequate nursing labor force during a pandemic, it must be ensured that policies and procedures include providing PPE and safeguarding the health of nurses and their families.(4)

On the one hundredth anniversary of the severe 1918 influenza pandemic, it was found that governments and health care systems from different countries are still inadequately prepared to face a health crisis of this magnitude and noted the need to implement coordinated intergovernmental tools and activities to improve the overall basic capacity to respond to global health threats.(5) It is precisely thus, how by late 2019 began one of the most complex pandemics known and which maintains global states and distinct government sectors concentrated on trying to control or mitigate the devastating effects of this pandemic.

At the date of publication of this editorial, Latin American countries will probably be suffering the most critical moments of the COVID-19 pandemic in which besides the health crisis, will highlight the inequality that has become evident in different countries globally and which are affecting public health and social wellbeing. As in other settings, this pandemic has changed all action axes of nursing, from the managerial role of nurses leading clinical teams, to health professionals, and those from academia, affecting nurses and nursing professors and researchers.

Under these conditions, health teams and nursing professionals are forming a first line in the units of critically ill patients, dealing with shortage of supplies and basic devices for care, including PPE. Many have undergone a reassignment of duties, have assumed new tasks, and have needed to modify their work systems by increasing the work load. In this regard, *in situ* evaluations of recent experiences have highlighted the need for clinical mobility as a way to increase the experience and improve skills and copacities within multidisciplinary teams that even permit a broader vision of the clinical and organizational panorama. Moreover, reducing the psychological impact in case of sudden reassignment to a different clinical environment.(6) However, independent of the greater or lesser experience, nursing professionals today confront not very encouraging results and evolution of patients and are witnesses to dilemmatic situations that become more frequent, generating distress within the teams.

In parallel, in a first line at community level, nurses face complex social and economic realities that prevent the population from adequately adopting contagion prevention and disease dissemination measures. This scenario exposes them, additionally, to the frustration and emotional involvement of the nursing contingent upon confirming the impossibility of helping people to recover or maintain optimal health. 

Regarding training contexts, the pandemic has limited classroom teaching and delayed clinical practices. Recommendations, and even requirements for quarantines and home confinement to ensure physical distancing have forced adopting a telework modality based on remote virtual teaching. This is where the question about whether universities have been sufficiently prepared to face these events is renewed and if they can effectively continue the teaching and education mission in a remote environment, far from the traditional campuses.(7) University work has moved to the homes and faces professors and students with new technology to at least develop the theoretical components of the study programs.(8) For its part, research has come to a halt, given the impossibility of carrying out field work and persist or have begun studies based on surveys on line or through telephone calls.

Paradoxically, everything negative and complex brought about by the coronavirus pandemic and which keeps Nursing at the limits of its capabilities and response contrasts with a resilient competency without reference, commitment, dedication, and effort that has led the members of the nursing staff to keep working, distancing themselves even from their own relatives and loved ones. 

With no desire to be less rigorous and still less to fall into professional complacency, I consider that there may be few qualifiers that describe the crucial task of Nursing within the health teams, as guarantors of oportune, safe, ethical and compassionate care. Today, it has been demonstrated that all the demands to shorten the gaps and provide an adequate standard of nurses for the distinct global care contexts are but the minimum requirements for communities to receive care and quality care. 

This 2020 will be remembered for a new pandemic, and probably there will be little celebration in this special year for Nursing. But six days from the bicentennial of Nightingale's birth, Banksy, the renowned anonymous artist left behind a remarkable piece titled Game Changer, with the message *"Thanks for all you’re doing. I hope this brightens up the place a bit, even if its only black and white."*(9) This artwork of art with profound and emotional sense of appreciation and gratitude to the health teams, taking the innocence of a child as its center, it has forever exalted nurses, and nursing itself, with the heroic courage with which it has confronted and will continue to confront situations, such as the current pandemic. 

**Figura 1:**
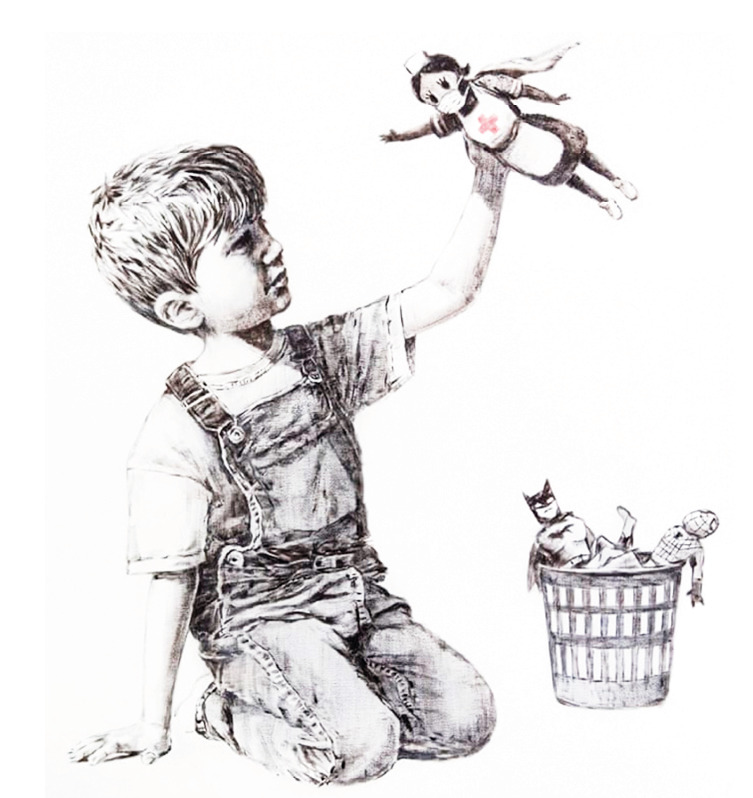
Source: Banksy's painting for the Southampton General Hospital called Game Changer**.**(10)
